# Unloader brace or high tibial osteotomy in the treatment of the young patient with medial knee osteoarthritis: a randomized controlled trial

**DOI:** 10.2340/17453674.2025.42846

**Published:** 2025-01-20

**Authors:** Mark STAM, Joost VERSCHUEREN, Mark V VAN OUTEREN, Reinoud W BROUWER, Robert D A GAASBEEK, Sorin G BLENDEA, Eline M VAN ES, Max REIJMAN, Sita M A BIERMA-ZEINSTRA

**Affiliations:** 1 Department of Orthopaedics and Sports Medicine, Erasmus MC University Medical Centre Rotterdam; 2 Department of Radiology & Nuclear Medicine, Erasmus MC University Medical Center Rotterdam; 3 Department of Orthopaedic Surgery, Martini Hospital, Groningen; 4 Department of Orthopedics and Traumatology, Meander Medical Center, Amersfoort; 5 Department of Orthopaedic Surgery, Franciscus Gasthuis & Vlietland Hospital, Rotterdam; 6 Department of General Practice, Erasmus MC University Medical Centre, Rotterdam, the Netherlands

## Abstract

**Background and purpose:**

For medial knee osteoarthritis (OA), operative and nonoperative treatment options are available. Two widely applied unloading therapies are a valgus unloader brace and a high tibial osteotomy (HTO). We aimed to compare the effects of a valgus unloader knee brace with an HTO on knee pain after 1 year in patients with symptomatic medial knee OA.

**Methods:**

We recruited patients from 9 Dutch hospitals between August 2014 and February 2019 for an open-labeled multi-center randomized controlled trial (Dutch Trial Register NL4200). Patients aged 18 to 65 years with symptomatic medial compartmental knee OA were randomized to either a valgus unloader brace or an HTO. The primary outcome was the pain subscale of the Knee injury and Osteoarthritis Outcome score (KOOS) after 1 year. Patients were evaluated at 3, 6, 9, 12, and 24 months.

**Results:**

51 patients were included in the study, of whom 23 were randomized to the unloader brace and 28 to the HTO. The HTO, compared with the unloader brace, showed a significant and clinically relevant difference at 12 months of follow-up in KOOS pain of –28 (95% confidence interval –43 to –13).

**Conclusion:**

We found that, on group level, an HTO is more effective in reducing knee pain than an unloader brace after 12 months.

In patients with knee osteoarthritis (OA), the medial knee compartment is more often affected than the lateral and patellofemoral compartment [1]. For those with knee OA unresponsive to nonoperative interventions, knee arthroplasty (KA) is a highly successful and commonly performed surgery [2]. However, KA is not the first treatment choice for young patients with medial knee OA, and first- and second-line treatment should be carried out before surgery [2,3]. For medial knee OA, operative and nonoperative treatment options are available that aim to unload the affected medial knee compartment before a KA [4-6]. These interventions aim to alter the biomechanics of the knee and consequently reduce symptoms [7,8]. Ideally, they revoke or postpone the need for a KA. Widely applied unloading therapies are a lateral wedge insole, a valgus unloader brace, and a high tibial osteotomy (HTO).

A valgus unloader brace is a popular nonoperative treatment option, with promising results regarding pain relief and improvement of function [9]. However, compliance appears to be a challenge [4,10].

An HTO intends to transfer the weight-bearing axis from the affected medial knee compartment to a slightly lateral position [8]. It has proven to be effective in reducing pain and functional symptoms [6,8,11]. Low conversion rates from HTO to KA have been found, with reported 10-year survival rates ranging from 73% to 98% [12,13]. Nonetheless, HTO is a technically demanding procedure with its inherent potential complications [6,14,15].

To date, no study has compared the effects of an unloader brace with an HTO in a randomized setting. The aim of this multi-center randomized controlled trial was to compare the effects on knee pain after 1 year of a valgus unloader knee brace with an HTO in patients between 18 and 65 years with symptomatic medial knee OA and varus malalignment. We hypothesize that an HTO would result in more alleviation of knee pain than an unloader brace.

## Methods

### Study design and setting

We conducted an open-labeled multi-center randomized controlled trial in patients with medial compartmental knee OA. The trial was carried out in 9 hospitals in the Netherlands, and patients were recruited between August 2014 and February 2019. Reporting follows the Consolidated Standards of Reporting Trials (CONSORT) guidelines.

### Participants

Patients between 18 and 65 years consulting an orthopedic surgeon in one of the participating centers for symptomatic medial knee OA were eligible to participate. Before visiting the orthopedic surgeon, patients were treated with nonoperative measures by their general practitioner, such as education, lifestyle changes, weight loss, exercise therapy, and pain medication. The criteria for inclusion were: knee pain located over the medial tibiofemoral compartment of the knee, knee pain for more than 3 months, with a severity of minimally 3 on a Numeric Rating Scale (NRS) (range 0 to 10), radiographic signs of medial knee OA with a Kellgren and Lawrence score of grade 1 to 3, and presence of varus malalignment with a maximum of 14° as measured on a whole-leg radiograph. Patients were required to have pain located over the medial tibiofemoral compartment to help ensure that the study population had clinical medial knee OA. A maximum varus malignment of 14° was chosen because in the participating hospitals it was common practice to perform a double osteotomy or use a bone graft in corrections greater than 14°. The inclusion criteria were in concordance with the Dutch National Guidelines on medial knee OA.

Patients were excluded when 1 of the following criteria was present: radiographic OA of the lateral compartment with a Kellgren and Lawrence score of grade 2 or higher, rheumatoid arthritis, grade 3 collateral ligament laxity, range of motion of < 100°, a flexion contracture of > 10°, history of fracture or previous open operation of the lower limb or lateral meniscectomy, past use of an orthopedic knee brace for knee OA in the same knee, contralateral HTO or brace if that knee has been included in this trial (thus, if both knees were symptomatic, the most affected knee was included), uncertainty concerning ability to attend the follow-up measurements, and insufficient understanding of the Dutch language, spoken and/or written.

To determine the patient’s eligibility, standing anteroposterior and lateral knee and long-leg radiographs were taken and assessed by the attending orthopedic surgeon of the participating hospital where the patient presented. The radiographs were used to measure the presence and severity of knee OA with the Kellgren and Lawrence score and the varus malalignment with the hip–knee–ankle (HKA) angle. The HKA angle was defined as the angle between 2 lines: 1 line from the center of the femoral head to the top of the femoral notch and a second line from the center of the ankle to the center of the tibial spines [16]. Patients were registered for the study by their own orthopedic surgeon and referred to the coordinating hospital (Erasmus MC University Medical Center) for enrollment and measurements. One specific researcher (EE) conducted all measurements. The actual treatment was provided at the patient’s own hospital.

### Randomization, blinding, and treatment allocation

Following informed consent and baseline measurements, patients were randomized to 1 of the 2 treatment groups in a 1:1 ratio. Randomization was stratified for experience of the orthopedic surgeon with performing an HTO procedure (more or less than 20 HTOs per year) and sex. The coordinating researcher contacted 1 researcher (not otherwise associated with the trial) who allocated treatment arms using computer-generated random numbers (central randomization). Microsoft Access was used for the randomization algorithm (Microsoft Corp, Redmond, WA, USA). The type of randomization was stratified balanced block randomization. Treatment arms were allocated in block sizes varying from 2 to 6.

The orthopedic surgeon and the patient were not blinded to the intervention.

### Interventions

#### Valgus unloader brace

Before the initiation of the RCT, an internal pilot study was performed to select the most appropriate brace for the trial. 3 widely available valgus unloader braces in the Netherlands were compared regarding comfort, convenience and pain relief in patients with medial knee OA from the orthopedic clinic of 1 of the participating centers. 9 patients, not participating in the RCT, wore each brace for 2 weeks. Based on their experiences, the Össur Unloader One brace (Össur hf., Reykjavík, Iceland) was chosen for its effect on pain reduction and its ease of use. The brace was fitted and customized to the patient’s knee by an orthotist at the start of the treatment. The brace had to be used according to the manufacturer’s instructions, meaning it needed to be worn for daily activities like standing, walking, exercising, and working throughout the day during the 2-year follow-up period.

Medication use was standardized for both groups and was given according to existing Dutch guidelines, according to the WHO analgesic ladder [17].

#### High tibial osteotomy

Patients received a medial open or lateral closed wedge HTO, according to the preferred surgical technique in the participating hospitals. The open wedge osteotomy was created through a medial approach to the proximal tibia by making a saw cut a few centimeters below the joint surface while preserving the lateral cortex. Subsequently, the saw cut was opened from the medial side, causing a valgus alignment of the lower leg as the lateral cortex acted like a hinge. The created open wedge was fixed on the medial side of the tibia with a titanium plate and screws (TomoFix, DePuy Synthes, PA, USA). In the case of the closed wedge osteotomy, the proximal tibia was approached through an anterolateral approach. The proximal tibia was cut a few centimeters below the joint surface while preserving the medial cortex. A second saw cut was made to create a bony wedge that was removed. The resulting wedge-shaped space was then closed, leading to valgus alignment of the lower leg as the medial cortex acted as a hinge. The anterior portion of the proximal part of the fibular head, which represents the anterior part of the proximal tibiofibular syndesmosis, was resected. No fibular osteotomy was performed. Subsequently, fixation was achieved using a titanium plate and screws (TomoFix, DePuy Synthes, PA, USA) or chrome-cobalt staples (Stepped High Tibial Osteotomy Staples, Stryker, MI, USA). The thickness of the wedge was calculated in advance to achieve the desired degree of correction. In both osteotomy techniques, fluoroscopy was used during the procedure to determine the position of the osteotomy planes and to monitor the degree of correction. The aim of both techniques was to create a valgus knee alignment of 4°.

The day after the operation, patients were mobilized with partial weightbearing on the operated on leg. Full weightbearing was allowed 2 weeks after surgery. Patients were discharged when they were able to walk without assistance, using 2 crutches, and with acceptable wound healing. After the initial postoperative mobilization, physiotherapy was recommended during the postoperative rehabilitation.

### Measurements

#### Primary outcome

Knee pain after 1 year of follow-up was assessed with the pain subscale of the Knee injury and Osteoarthritis Outcome score (KOOS). The KOOS questionnaire consists of 5 subscales: pain, symptoms, activities of daily living (ADL), sports, and quality of life (QoL) [18]. A score is calculated for each subscale, which ranges from 0 to 100, with 100 being the optimal score.

#### Secondary outcomes

Knee pain was assessed with the KOOS pain subscale after 24 months, the Numeric Rating Scale (NRS) for pain severity [19], other subscales of the KOOS, the Intermittent and Constant Osteoarthritis Pain score (ICOAP) [20], and the Hospital for Special Surgery scale (HSS) [21]. In addition, painkillers, brace use, self-reported complaints, and (serious) adverse events were evaluated during follow-up by patient-completed questionnaires and medical records. The type of painkillers used (paracetamol, NSAIDs, and opioids), was recorded.

NRS pain ranged from 0 to 10, where 0 represented no pain [19]. The ICOAP is a questionnaire comprising 11 items concerning intermittent and constant knee pain, which is converted into a pain score that ranges from 0 to 44, with 0 representing no pain [20]. HSS, which was conducted by the researcher, is a scale with subscores relating to pain, range of motion, instability, flexion deformity, alignment, leg extension, and medical aids, which add up to a total score with a maximum of 100 points representing no knee complaints [21].

Adverse events were self-reported by the patient with questionnaires during follow-up. Serious adverse events were registered by the participating centers. All reported complications and re-interventions that could have been objectified and reasonably have been a consequence of the given treatment were analyzed as (serious) adverse events.

Patients completed all questionnaires digitally at baseline and 3, 6, 9, 12, and 24 months after randomization, except for the KOOS questionnaire. The KOOS questionnaire was filled in at baseline, 12, and 24 months. Study data were collected and managed using GemsTracker electronic data capture tool hosted at the Erasmus MC [22].

All included patients visited the coordinating hospital at baseline and after 1 year of follow-up for a physical examination for the HSS rating scale.

### Sample size

When we calculated the sample size, no studies on minimal clinically important difference (MCID) for the KOOS score were available. We based our initial sample size calculation on detecting a difference with an effect size of 0.5 in favor of a surgical intervention compared with a nonoperative strategy, with 80% power and a 2-side type 1 error of 5%. To accommodate a potential loss to follow-up of 15% over 1 year, the target sample size was set to 124 patients (62 per group).

However, the study experienced a delay because of problems in recruiting patients willing to be randomized to surgical treatment or non-surgical treatment. In agreement with the grant supplier and the Dutch Orthopaedic Association, we recalculated the required sample size based on the standard deviation (SD) of the KOOS pain subscale, using the baseline data of our own study. The observed SD of 15.7 was considerably smaller than the estimated SD of 22 used in the initial calculation, leading to a redefinition of the required sample size. We determined that 28 participants per group (a total of 56) would be sufficient, with the aim to enroll 64 patients, allowing for a potential dropout rate of up to 15% over a 1-year period. Finally, the recruitment of patients was finished in 2019 in agreement with the grant supplier.

### Statistics

An intention-to-treat analysis was performed. To answer our primary research question, we used a linear regression model with KOOS pain subscale after 1 year as dependent variable, adjusted for age, sex, surgeon’s experience, and KOOS pain at baseline. Missing values were handled with listwise deletion. We checked the following model assumptions: linearity, multicollinearity, homoscedasticity, and normality and independence of residuals in the linear regression model. None of the assumptions were violated. A linear mixed model analysis was used to assess the secondary outcomes. We used an unstructured covariance structure and a restricted maximum likelihood (REML) model for estimation. The fixed factors added to the model were the interaction term of time by treatment (the multiplication follow-up and randomization), age, sex, and experience of the surgeon. The model assumptions of linearity, homoscedasticity, and normality of residuals were assessed and considered not violated. The estimated marginal means from the linear mixed model were presented. 95% confidence interval (CI) was reported. IBM SPSS statistics was utilized for all analyses (IBM Corp, Armonk, NY, USA).

### Ethics, registration, data sharing, use of AI, funding, and disclosures

The Erasmus MC University Medical Center ethics committee approved the research protocol, and all patients gave written informed consent. The trial was registered in the Dutch Trial Register prior to the inclusion of the first subject (NTR number NL4200). This study was financially supported by the Dutch Arthritis Foundation (Reuma Nederland; https://trialsearch.who.int/Trial2.aspx?TrialID=NL-OMON27146).

No artificial intelligence tools were used for this study. The data that supports the findings for this study is available to other researchers from the corresponding author upon reasonable request. Complete disclosure of interest forms according to ICMJE are available on the article page, doi: 10.2340/17453674.2025.42846

## Results

### Patients

The exact number of potentially eligible patients at the participating centers was undetermined. Of the 107 patients enlisted for the study, 21 strongly preferred brace treatment and 21 patients strongly preferred the osteotomy. These patients were unwilling to be randomized and were therefore excluded. 7 patients did not meet our inclusion criteria and another 7 patients refrained from treatment. This resulted in a final study population of 51 patients. 23 patients were randomized to the brace and 28 to the HTO. On average, brace treatment was started 24 days (SD 18) and surgery was performed 74 days (SD 60) after inclusion. The response rate of 1-year follow-up of KOOS pain score was 91% for the brace and 96% for the HTO group.

After randomization, 3 patients (13%) from the brace group crossed over to the HTO group, 1 patient before the 12 months’ time point and 2 patients between the 12 and 24 months’ time point. 3 patients (11%) in the HTO group did not receive an HTO. In 1 patient, the treating clinician noted prominent patellofemoral knee osteoarthritis on the additional MRI and SPECT-CT and deemed the patient not suitable for an HTO. 2 patients chose not to proceed with the surgery after they had been randomized. 3 patients (13%) in the brace group were converted to a unicompartmental knee arthroplasty (UKA) or a total knee arthroplasty (TKA) and 1 patient (4%) in the HTO group was converted to a TKA during the 24 months’ follow-up. Detailed information can be found in the Figure.

**Figure:**
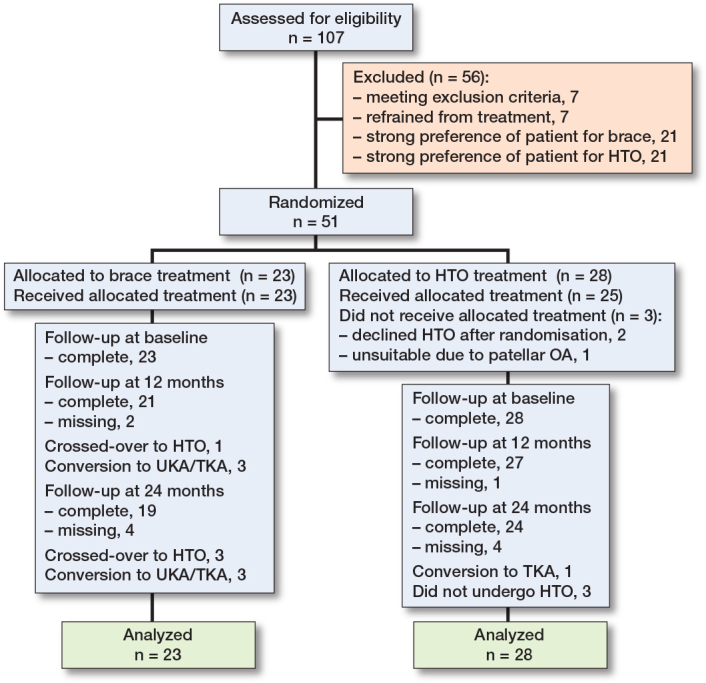
Flowchart of patients.

Table 1 provides information on the baseline characteristics of the included patients. 88% of patients had experienced symptoms for over 6 months before enrollment. Age and KOOS pain score at baseline differed between the brace and HTO group. Patients in the brace group were on average 5.2 years younger (49.9 vs 55.1 years of age) and scored 7 points higher on the KOOS pain scale at baseline (43 vs 36). The varus angle ranged from 1° to 13°.

**Table 1:** Baseline characteristics of study population. Values are mean (standard deviation) or count

Factor	Brace (n = 23)	HTO (n = 28)
Age	49.9 (6.8)	55.1 (6.5)
Male sex, n	14	17
BMI	29.0 (4.1)	29.8 (4.4)
Left leg affected, n	10	15
Varus angle, °	6.7 (3.1)	5.7 (2.3)
Paid work, n	15	21
Months of symptoms, n		
1–3	1	0
3–6	2	3
6–12	7	11
> 12	13	14
KOOS pain	43 (3.0)	36 (2.7)
HSS	73 (12)	72 (7.8)
NRS-pain at rest	5.4 (2.2)	5.4 (2.1)
NRS-pain during activity	7.6 (1.8)	7.9 (1.6)
ICOAP total	23 (9.4)	25 (7.3)
K&L medial compartment	2.5 (0.5)	2.6 (0.5)
K&L lateral compartment	0.7 (0.5)	0.8 (0.4)

KOOS = Knee Osteoarthritis Outcome Score.

HSS = Hospital for Special Surgery Knee-Rating Scale.

NRS = Numeric Rating Scale.

ICOAP = Intermittent and Constant Osteoarthritis Pain score.

K&L = Kellgren and Lawrence score.

At 12 months, 55% of patients in the brace group complied with the treatment. The knee brace was worn for 6.4 hours (SD 6.9) per day on average.

### Primary outcome

The adjusted estimated mean KOOS pain score at 12 months’ follow-up for patients allocated to the brace was 49 (CI 44–54) and 71 (CI 67–74) for patients allocated to the HTO (Table 2). The improvement in KOOS pain scores at 12 months’ follow-up was significant larger in the HTO group (35, CI 31–38) compared with the brace group (5.8, CI 2.0–9.5).

**Table 2:** Primary outcome of patients randomized to a brace or HTO. Time is measured from inclusion date. Values are unstandardized predicted means and regression coefficients with 95% confidence interval in parentheses

KOOS pain	Brace (n = 23)	HTO (n = 28)	Between-group difference
At 12 months Improvement during	49 (44–54)	71 (67–74)	–28 (–43 to –13)
first 12 months	5.8 (2.0–9.5)	35 (31–38)	–29 (–44 to –15)

KOOS = Knee Osteoarthritis Outcome Score.

KOOS pain was known for 48/51 patients at 12 months.

KOOS pain was adjusted for age, sex, surgeon’s experience, and

KOOS pain at baseline.

### Secondary outcomes

All secondary outcomes showed a statistically significant difference in improvement between the 2 groups in favor of the patients allocated to the HTO group (Table 3, see Appendix).

**Table 3:** Secondary outcomes of patients randomized to a brace (n = 23) or HTO (n = 28) during 2-year follow-up. Values are adjusted mean estimate with 95% confidence interval in parentheses

Factor	T0	T3	T6	T9	T12	T24
KOOS, Pain						
Brace	43 (37 to 49)	n/a	n/a	n/a	49 (38 to 60)	51 (40 to 63)
HTO	35 (29 to 40)	n/a	n/a	n/a	70 (60 to 80)	72 (61 to 82)
Difference**^a^**	8.2 (0.0 to 16)	n/a	n/a	n/a	–21 (–36 to –5.7)	–20 (–36 to –4.4)
KOOS, Symptoms						
Brace	50 (43 to 58)	n/a	n/a	n/a	57 (46 to 67)	56 (46 to 66)
HTO	44 (38 to 51)	n/a	n/a	n/a	67 (58 to 76)	69 (60 to 77)
Difference**^a^**	6.0 (–4.2 to 16)	n/a	n/a	n/a	–11 (–25 to 3.5)	–13 (–26 to 0.7)
KOOS, ADL						
Brace	46 (39 to 53)	n/a	n/a	n/a	56 (44 to 68)	58 (47 to 69)
HTO	44 (38 to 51)	n/a	n/a	n/a	69 (58 to 79)	73 (64 to 83)
Difference**^a^**	2.0 (–7.6 to 12)	n/a	n/a	n/a	–12 (–29 to 3.8)	–16 (–31 to –0.9)
KOOS, Sports and recreation						
Brace	18 (8.7 to 27)	n/a	n/a	n/a	20 (6.2 to 33)	31 (16 to 46)
HTO	14 (5.4 to 22)	n/a	n/a	n/a	39 (28 to 51)	43 (30 to 56)
Difference**^a^**	4.4 (–8.2 to 17)	n/a	n/a	n/a	–20 (–38 to –1.8)	–12 (–32 to 8.6)
KOOS, QoL						
Brace	26 (20 to 33)	n/a	n/a	n/a	31 (21 to 42)	38 (26 to 50)
HTO	24 (18 to 29)	n/a	n/a	n/a	50 (40 to 60)	56 (45 to 67)
Difference**^a^**	2.6 (–6.3 to 12)	n/a	n/a	n/a	–19 (–33 to –3.7)	–18 (–34 to –1.3)
NRS–pain at rest						
Brace	5.4 (4.5 to 6.4)	4.7 (3.5 to 5.8)	5.5 (4.4 to 6.6)	5.1 (4.0 to 6.3)	4.4 (3.4 to 5.4)	4.6 (3.3 to 5.8)
HTO	5.3 (4.5 to 6.2)	3.9 (2.8 to 5.0)	2.8 (1.8 to 3.8)	2.6 (1.5 to 3.6)	2.3 (1.4 to 3.1)	2.4 (1.3 to 3.4)
Difference**^a^**	0.1 (–1.2 to 1.4)	0.7 (–0.8 to 2.4)	2.7 (–4.2 to –1.2)	2.6 (1.0 to 4.1)	2.1 (0.8 to 3.5)	2.2 (0.6 to 3.8)
NRS–pain during activity						
Brace	7.7 (6.9 to 8.4)	5.6 (4.4 to 6.7)	6.2 (5.0 to 7.4)	6.1 (4.9 to 7.3)	6.5 (5.3 to 7.7)	6.2 (4.9 to 7.6)
HTO	7.9 (7.3 to 8.6)	6.0 (5.0 to 7.0)	4.8 (3.8 to 5.9)	4.3 (3.2 to 5.3)	3.7 (2.7 to 4.8)	3.3 (1.7 to 4.8)
Difference**^a^**	–0.2 (–1.3 to 0.8)	–0.4 (–1.9 to 1.1)	1.4 (–0.2 to 3.0)	1.9 (0.2 to 3.4)	2.8 (1.2 to 4.4)	3.0 (1.1 to 4.8)
HSS						
Brace	72 (68 to 76)	n/a	n/a	n/a	78 (72 to 83)	n/a
HTO	72 (68 to 75)	n/a	n/a	n/a	86 (81 to 91)	n/a
Difference**^a^**	0.7 (–4.9 to 6.4)	n/a	n/a	n/a	–8.5 (–16 to –0.9)	n/a
ICOAP, Intermittent						
Brace	13 (11 to 15)	13 (10 to 15)	11 (8.2 to 14)	12(8.7 to 14)	11(8.4 to 14)	11 (7.8 to 14)
HTO	14 (12 to 16)	11 (8.5 to 13)	9 (6.5 to 11)	8.4 (6.0 to 11)	7.1 (4.8 to 9.5)	5.9 (3.3 to 8.4)
Difference**^a^**	–0.8 (–3.5 to 2.0)	2.1 (–1.1 to 5.3)	2.0 (–1.6 to 5.7)	3.1 (–0.7 to 6.9)	3.9 (0.3 to 7.5)	4.9 (1.0 to 8.8)
ICOAP, Constant						
Brace	9.8 (7.9 to 12)	8.8 (6.6 to 11)	8.2 (5.7 to 11)	8.6 (6.0 to 11)	8.3 (6.0 to 11)	7.7 (5.2 to 10)
HTO	11 (9.6 to 13)	8.2 (6.2 to 10)	5.9 (3.7 to 8.0)	6.5 (4.2 to 8.8)	5.1 (3.0 to 7.1)	4.6 (2.4 to 6.7)
Difference**^a^**	–1.5 (–4.0 to 1.0)	0.7 (–2.4 to 3.7)	2.3 (–1.0 to 5.7)	2.1 (–1.4 to 5.6)	3.3 (0.1 to 6.4)	3.2 (–0.2 to 6.5)
Total						
Brace	23 (20 to 27)	22 (17 to 26)	19 (14 to 24)	20 (15 to 26)	20 (15 to 24)	19 (13 to 24)
HTO	25 (22 to 29)	19 (15 to 23)	15 (10 to 19)	15 (10 to 20)	12 (7.8 to 17)	10 (5.8 to 15)
Difference**^a^**	–2.1 (–7.1 to 2.9)	2.9 (–3.2 to 9.0)	4.5 (–2.3 to 11)	5.3 (–1.7 to 12)	7.3 (0.7 to 14)	8.1 (0.9 to 15)

A linear mixed model was utilized for the analysis. Outcomes were adjusted for age, sex, and surgeon’s experience

T indicates time in months after inclusion; n/a = not applicable; For other abbreviations, see Table 1.

a Difference between Brace and HTO

### Adverse events

17/23 patients treated with a brace reported a total of 21 complaints (Table 4). The most frequent complaints concerning the brace were skin irritation (16 patients) and numbness (5 patients).

**Table 4:** Adverse events of patients treated with a brace or HTO during 2-year follow-up. Values are count

Randomized as:	Brace (n = 23) ^a^		HTO (n = 28) ^b^
Treated as:	Brace	HTO	HTO
	(n = 23)	(n = 3)	(n = 25)
Patients with complaints	16	1	19
Overall complaints	21	1	27
Patient-reported complaints			
Skin irritation	16	0	0
Irritation	0	0	12
Numbness	5	1	11
Wound infection	0	0	3
Post-surgery bleeding	0	0	1
Reoperations			
Conversion to TKA/UKA	3	0	1
Plate removal	–	0	10
Reoperation for wound infection	–	0	1

a 3 patients crossed over to HTO.

b 3 patients did not undergo an HTO.

TKA = total knee arthroplasty.

UKA = unicompartmental knee arthroplasty

In comparison, 19 of the 25 treated patients in the HTO group reported a total of 27 complaints. Irritation (12 patients) and numbness (11 patients) were the most common complaints. Other self-reported complaints were wound infection (3 patients) and post-surgery bleeding (1 patient).

10 of the 25 treated patients in the HTO group underwent plate removal during the course of the study. The average duration from HTO to plate removal was 8.8 months (SD 2.5). 6 plates were removed before the 12 months’ time point and 4 plates were removed between 12 and 24 months. Finally, 3 patients were converted to a TKA/UKA before the 12 months’ time point. In the HTO group, 1 patient was converted to a TKA between 12 and 24 months. The average duration from the start of the brace treatment to conversion to a TKA/UKA was 7.7 months (SD 1.2) and the duration from HTO to conversion to a TKA was 12 months. In the HTO group, 1 patient received an HTO for the contralateral knee during follow-up.

### Painkiller use

A clear decrease in painkiller use was seen in the HTO group at 12 and 24 months (Table 5).

**Table 5:** Painkiller use of patients randomized to a brace or HTO during 2-year follow-up. Values are count

Brace/HTO	T0	T3	T6	T9	T12	T24
Brace, n	23	21	18	16	20	17
No painkillers	13	15	13	8	10	9
Paracetamol	4	3	1	1	4	6
NSAID	4	1	2	5	4	2
Opioid	2	2	2	2	2	0
HTO, n	28	25	24	22	27	26
No painkillers	7	3	9	12	19	20
Paracetamol	9	5	8	5	4	2
NSAID	11	10	4	3	3	2
Opioid	1	7	3	2	1	2

T indicates time in months after inclusion.

Painkiller use was assessed by patient-completed questionnaires.

The painkiller with the highest analgesic potency used is shown.

NSAID = non-steroidal anti-Inflammatory drugs: diclofenac, etoricoxib, ibuprofen, or naproxen.

Paracetamol = acetaminophen.

Opioid = oxycodone or tramadol.

## Discussion

The primary aim of this randomized controlled trial was to compare the effects on knee pain of an unloader brace with an HTO in patients with medial knee OA. The results of this study showed that on group level an HTO is more effective in reducing knee pain compared with an unloader brace.

The difference in improvement between the brace and HTO group for KOOS pain during the first 12 months was almost double the minimal clinically important difference (MCID) for KOOS pain of 15.4 [23]. We found an improvement in the brace group for KOOS pain of 5.8 after 1 year. This improvement was similar to earlier studies, reporting changes on the KOOS pain subscale after 1 year of 7.6 and 7.7 [9,10]. In the HTO group, we found an increase from baseline to 1 year of follow-up for KOOS pain of 35. This is in accordance with previous studies [8,11]. De Pieri et al. [8] reported a median change in KOOS pain of 31.9 and Jacquet et al. [11] found an improvement after 1 year of 35.

The difference in effectiveness between the interventions could be partially explained by the poor compliance in the brace group. Nonetheless, this study provides a realistic assessment of the brace’s performance in everyday clinical practice where patient adherence may be inconsistent.

Patients undergoing HTO usually have a postoperative treatment and recovery period lasting up to 6 months for most of the patients [24]. Our findings indicate that patients who underwent HTO already show significant symptom improvement at timepoints before the 1-year follow-up. This study demonstrates that HTO could rapidly improve pain and function for younger patients with medial knee OA.

Noteworthy were the 3 crossovers and the 3 conversions to TKA/UKA in the 23 patients allocated to brace treatment. This high percentage of patients in the brace group switching to a different treatment might be attributable to ineffectiveness of the brace [4].

In both groups, negative effects of the treatment were experienced. Many patients allocated to the brace complained about skin irritation and/or numbness when wearing the brace. This is supported by previous research that recorded skin irritation, bad fit, and discomfort caused by the brace [4,5,9]. The discomfort while wearing the brace, in combination with minimal treatment effect of the brace, impedes therapy compliance, according to the literature [4,10]. Similar to other surgical procedures, HTO carries risks. Irritation due to hardware material was frequently reported in our study, which necessitated plate removal in 40% of the HTO patients during the course of the study. In addition, wound infections were recorded, with 1 patient requiring reoperation. Documented complications in earlier studies included hardware failure, intraoperative fracture of the lateral bone, infection, loss of correction, nerve injury, and nonunion [6,14,15]. This emphasizes the importance of considering adverse events during treatment decision-making.

### Strengths

Our internal pilot study, in which we selected the most appropriate brace regarding comfort, convenience, and pain relief, ensured the best possible brace comparator for the HTO.

### Limitations

The target sample size was not reached due to multiple difficulties encountered during enrollment. First, numerous patients expressed a strong preference for one or other of the treatments and consequently refused randomization. Second, the considerable travel distance from a recruiting center to the coordinating hospital deterred some patients from participating. Third, the takeover and subsequent policy change by potentially 1 of the largest recruiting centers resulted in a diminished pool of potential study candidates. This high-volume center ceased operations, and the HTO-performing surgeons were dispersed to various other hospitals. Fourth, more than the 107 enlisted patients were approached by the treating orthopedic surgeons to participate, but European privacy regulations (General Data Protection Regulation) prevented us from obtaining information concerning these eligible patients.

Another limitation was a potential random sampling error due to baseline differences in age and KOOS pain, which we adjusted for in the primary outcome analysis. Frequent crossovers and conversions to TKA/UKA might have led to an overestimation of the brace’s treatment effect, as supported by the as-treated results (Table 6, see Appendix). For that reason, the as-randomized results should be interpreted with caution. Also, the type of intervention did not allow blinding, potentially contributing to a larger placebo effect in patients allocated to HTO. In addition, the HTO group lacked a standardized procedure due to the performance of open and closed wedge osteotomies, and the variation in surgical techniques and surgeons among the participating centers. However, a previous study reported no clinically relevant differences between open and closed HTO [25], and randomization was stratified for surgeon’s experience. Lastly, no restrictions on painkiller use were imposed before completing the pain questionnaires. As the brace group used more painkillers at 12 and 24 months, painkiller use cannot have caused the differences in outcome.

**Table 6:** Primary outcome of patients treated with a brace or HTO (sensitivity analysis). Excluded from this sensitivity analysis were patients who did not undergo HTO (n = 3), crossed over to HTO (n = 1), or received TKA/UKA (n = 3) before the 12 months’ time point. Values are unstandardized predicted means and regression coefficients with 95% confidence interval in parentheses

Factor	Brace (n = 19)	HTO (n = 25)	Between-group difference
KOOS pain after 12 months	49 (44–54)	73 (68–78)	–36 (–21 to –51)

Also see Footnote for Table 2.

### Conclusion

HTO is more effective in alleviating knee pain after 1 year compared with a brace. The high number of conversions to TKA/UKA and crossovers to HTO questions the effectiveness of the brace as well. Based on these results, HTO appears more successful in achieving the treatment objectives than the brace.

## Notes

MS: collection, assembly, analysis, and interpretation of the data, and drafting of the article. JV: conception and design of the work, collection, assembly, analysis, and interpretation of the data, and drafting of the article. MO: conception and design of the work, collection and assembly of the data, and critical revision of the article for important intellectual content. RB, RG, SB: provision of patients, and critical revision of the article for important intellectual content. EE: administrative and logistic support, collection, assembly, analysis, and interpretation of the data, drafting of the article, and critical revision of the article for important intellectual content. MR: conception and design of the work, administrative and logistical support, collection, assembly, analysis, and interpretation of the data, drafting of the article, and critical revision of the article for important intellectual content. SBZ: conception and design of the work, obtaining of funding, and critical revision of the article for important intellectual content.

The authors would like to thank prof. dr. Dimitris Rizopoulos, head of the department of biostatistics of the Erasmus MC, for his expert advice on the statistical analysis. They would also like to express their appreciation to the patients, the participating centers, and medical doctors for their cooperation during the study.

Handling co-editors: Li Felländer-Tsai and Philippe Wagner

*Acta* thanks Margareta Hedström and Aleksi Reito for help with peer review of this manuscript.
